# Antibacterial and Proangiogenic Hydrogel Microneedle Patches for Wound Healing

**DOI:** 10.1002/smmd.70014

**Published:** 2025-09-09

**Authors:** Junyi Zhang, Yunjie Shi, Yixin Zhang, Zhiju Fang, Yechao Zhou, Feika Bian, Yuyang Zhang, Weijian Sun

**Affiliations:** ^1^ Department of Colorectal and Anal Surgery The First Affiliated Hospital of Wenzhou Medical University Wenzhou China; ^2^ Department of Gynecology The First Affiliated Hospital of Wenzhou Medical University Wenzhou China

**Keywords:** antibacterial, drug delivery, hydrogels, microneedle, wound healing

## Abstract

Wounds represent a global and challenging healthcare issue, resulting in a cascade of consequences. Despite the widespread application of existing wound dressings, their performance and efficacy are significantly limited in terms of biocompatible matrices and functionalization for promoting vascularization and antimicrobial activity. In this study, we propose a drug‐loaded microneedle based on a copolymer hydrogel composed of methacrylated chitosan and polyethylene glycol diacrylate incorporating antimicrobial peptide and vascular endothelial growth factor. These microneedles were applied to wounds where their degradation facilitated the release of the loaded drugs to exert antibacterial and angiogenic effects. In vitro experiments demonstrated that our microneedles exhibit uniform morphology, good structural integrity, controlled drug release, and other excellent properties. Upon interaction with cells and bacteria, they displayed biocompatibility and superior dual antibacterial capabilities. In an in vivo infectious wound model, the microneedles significantly promoted wound healing through their antibacterial and angiogenic effects, showing clear advantages over the control group. Thus, these drug‐loaded microneedles serve as a multifunctional dressing, offering a promising novel strategy for wound repair.

## Highlights

- Dual‐functional microneedles with AMP and VEGF promote wound healing.
- Show strong antibacterial, angiogenic, and biocompatible properties.
- Achieve controlled drug release and reduce inflammation in vivo.
- Significantly accelerate healing in infected diabetic wounds.

## Graphical abstract

A photo‐crosslinkable pre‐gel composed of CS‐MA, PEGDA, AMP, and VEGF was fabricated into biodegradable microneedles via mold casting and UV curing. Upon application to infected wounds, the microneedles gradually degrade, enabling sequential release of AMP and VEGF to achieve antibacterial and proangiogenic effects, thereby promoting efficient wound healing.

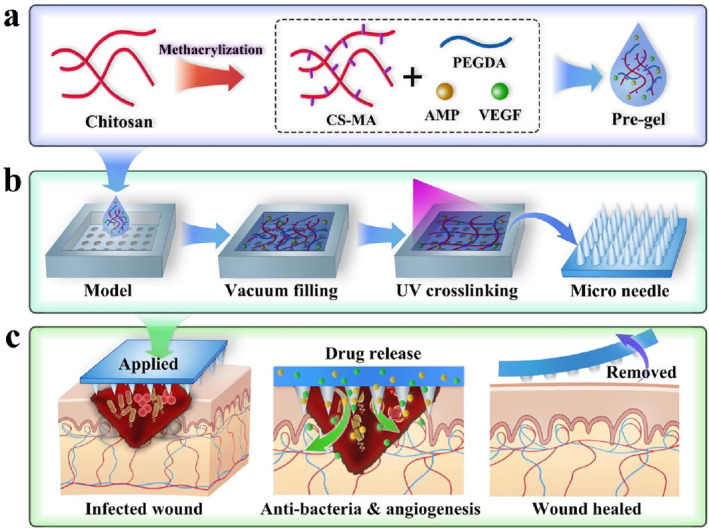

## 1 Introduction

Wounds are a global and challenging healthcare issue, leading to a series of consequences such as local infections and tissue necrosis [1, 2]. If not properly managed, they can even cause life‐threatening conditions like sepsis and organ failure. Although existing wound dressings, such as ointments, gauze, and synthetic polymer patches, have been widely used in wound repair, their performance and efficacy have significant limitations [3, 4, 5]. For instance, traditional dressings often lack water content and flexibility, making them mechanically and biologically incompatible with skin tissue [6, 7, 8, 9]. Clinical ointments or gels, on the other hand, are often non‐formable, making prolonged interaction with wounds difficult [10, 11]. Moreover, most dressings primarily provide passive protection, lacking drug delivery functions, crucial antibacterial and angiogenic capabilities [12, 13]. Appropriate blood circulation is fundamental to wound healing as it provides the wound with adequate nutrients and oxygen, promoting cell proliferation and repair. Meanwhile, antibacterial action reduces the risk of infection, creating a favorable microenvironment for healing. Therefore, a functionalized dressing with a biocompatible matrix for wound repair is highly anticipated.

**FIGURE 1:**
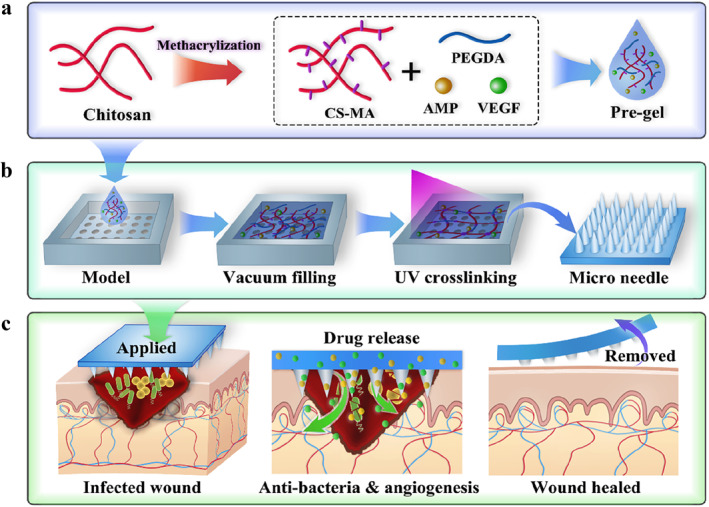
(a) Synthesis of CS‐MA and preparation of the pre‐gel. Chitosan was modified via methacrylation to form CS‐MA, which was then blended with PEGDA, AMP, VEGF, and a photoinitiator to create a photocrosslinkable pre‐gel. (b) Microneedle fabrication. The pre‐gel was vacuum‐filled into a microneedle mold, UV‐cured, and demolded to obtain drug‐loaded microneedles. (c) Application in infected wound healing. The microneedles were applied to the wound site where hydrogel degradation enabled sequential release of AMP (antibacterial action) and VEGF (proangiogenic effect), synergistically accelerating wound healing.

Herein, we propose microneedles with antibacterial and angiogenic functions for wound healing (Figure 1). Microneedles are a novel drug delivery system that can penetrate the skin's surface and deliver drugs directly to the target site, improving drug bioavailability and therapeutic outcomes [14, 15]. However, many current microneedle systems still face significant limitations. Metal microneedles, while mechanically robust, often suffer from poor biocompatibility and risk of tissue irritation. Polymer‐based microneedles generally offer better biocompatibility but typically lack intrinsic antibacterial properties and exhibit limited drug loading versatility, often restricted to either small molecules or hydrophilic compounds. Moreover, the mechanical properties of conventional microneedles are often fixed and not tunable to match varying therapeutic needs. As a material for microneedles, hydrogels provide a soft and moist matrix suitable for biological tissues, resembling the extracellular matrix and facilitating cell proliferation [16, 17, 18, 19]. Among these, chitosan (CS) derived hydrogels have excellent antibacterial properties, biodegradability, and biocompatibility, making them an ideal biomedical material [20, 21]. As for the drugs loaded onto the microneedles, antimicrobial peptides (AMP) exhibit significant antibacterial activity, preventing infection and creating a conducive healing environment [22, 23]. Meanwhile, vascular endothelial growth factor (VEGF) promotes angiogenesis, accelerates local blood circulation, enhances nutrient and metabolic supply, and expedites wound healing [24, 25, 26]. Therefore, designing chitosan‐derived microneedles loaded with AMP and VEGF is expected to achieve dual effects of antibacterial and angiogenic functions, thereby effectively promoting wound repair. However, such wound dressings have rarely been reported.

In this study, we propose a drug‐loaded microneedle based on a copolymer hydrogel of methacrylated chitosan (CS‐MA) and polyethylene glycol diacrylate (PEGDA) incorporating antibacterial AMP and angiogenic VEGF. Specifically, chitosan side chains were modified with methacrylate to form CS‐MA with photocrosslinking capabilities. This polymer was then mixed with PEGDA to serve as the primary component of the pre‐gel. After doping with AMP and VEGF, the pre‐gel was filled into a microneedle mold under vacuum mediation and cured under UV irradiation to form microneedles. The microneedles were then applied to wounds where their degradation released the loaded drugs to exert antibacterial and angiogenic effects. In vitro experiments demonstrated that our microneedles have uniform morphology, good structural integrity, controlled drug release, and other excellent properties. When interacting with cells and bacteria, they exhibited biocompatibility and superior dual antibacterial capabilities. In an in vivo infectious wound model, the microneedles significantly promoted wound healing through antibacterial and angiogenic effects, showing clear advantages over the control group. Thus, these drug‐loaded microneedles serve as a multifunctional dressing, providing a promising new strategy for wound repair.

## 2 Results and Discussion

**FIGURE 2:**
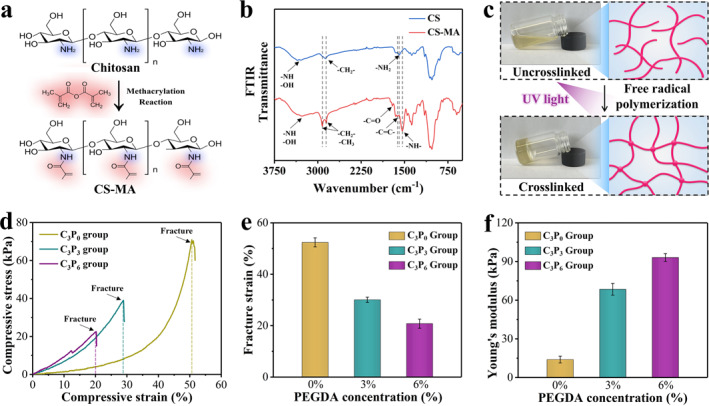
Preparation and characterization of CS‐MA hydrogel. (a) Schematic diagram of CS‐MA synthesis. (b) FTIR spectra of CS and CS‐MA. (c) Digital photos and schematic diagrams of CS‐MA hydrogel before and after crosslinking. (d) Compression curves of various CS‐MA hydrogels. (e) Fracture strain of various CS‐MA hydrogels. (f) Young's modulus of various CS‐MA hydrogels.

In a typical experiment, in order to obtain CS‐MA, we dissolve CS in an acidic solution, and then perform an esterification reaction with methacrylic anhydride to introduce MA groups on its side chains to obtain photocrosslinkable CS‐MA product (Figure 2a and Supporting Information S1: Figure S1). This occurs because the ‐NH_2_ and ‐OH groups on the CS molecular chain, due to their strong nucleophilicity, attack the carbonyl group of methacrylic anhydride, ultimately forming amide and ester bonds. This reaction successfully introduces the photosensitive methacrylate group into the CS backbone endowing it with the ability to crosslink under ultraviolet light.

As shown in Figure 2b, the Fourier transform infrared (FTIR) spectra of CS and CS‐MA further confirm that the reaction proceeded as expected. In the FTIR spectrum of CS, the broad peak at 3400 cm^−1^ is attributed to the stretching vibrations of ‐OH and ‐NH_2_ groups. The sharp peak at 2870 cm^−1^ corresponds to the symmetric stretching vibrations of ‐CH_2_‐, and the peak at 1580 cm^−1^ is due to the bending vibrations of ‐NH_2_. After the reaction with methacrylic anhydride, the FTIR spectrum of CS‐MA still shows a broad peak at 3400 cm^−1^, indicating the presence of unreacted ‐OH and ‐NH_2_ groups. A new peak at 2920 cm^−1^ appears, which is assigned to the asymmetric stretching vibrations of ‐CH_3_ in the methacrylate group. The significant absorption peak at 1620 cm^−1^ confirms the introduction of the C=C bond. Notably, the intensity of the ‐NH_2_ peak at 1580 cm^−1^ in CS decreases and a new peak at 1530 cm^−1^ appears, which is attributed to the bending vibrations of ‐NH‐, confirming the formation of amide bonds. Additionally, the weak absorption peak near 1720 cm^−1^ is due to the stretching vibrations of C=O, which is related to the formation of ester bonds. These spectral changes confirm that the methacrylate groups were successfully grafted onto the CS molecular chain through amide and ester bonds.

To verify its photocrosslinking ability, CS‐MA solution was mixed with a photoinitiator in a glass vial (Figure 2c). Before UV irradiation, the solution was free‐flowing. After exposure to UV light, it rapidly solidified into a shape‐defined covalent hydrogel. This happens because, on a microscopic scale, photo‐triggered radical polymerization enables linear CS‐MA chains to crosslink via C=C bond cleavage and reorganization of the methacrylate groups, forming a three‐dimensional hydrogel network. This property is beneficial for microneedle fabrication.

To optimize the hydrogel's properties, PEGDA was added to the synthesized CS‐MA to increase crosslinking density. The composite hydrogel was formed by UV‐initiated copolymerization. During this process, the methacrylate groups of CS‐MA and the diacrylate groups of PEGDA underwent free‐radical polymerization, forming a new 3D network. Based on factors like pre‐gel solubility, viscosity, and gel water content, three PEGDA concentrations were chosen. The CS‐MA concentration was fixed at 3% (w/v) with PEGDA concentrations of 0%, 3%, and 6% (w/v), labeled as C_3_P_0_, C_3_P_3_, and C_3_P_6_, respectively. Theoretically, increasing the PEGDA content introduces more crosslinking sites, thus increasing crosslinking density and potentially enhancing material rigidity.

The mechanical properties of the three hydrogel groups were assessed via compression tests (Figure 2d). The stress‐strain curves revealed that C_3_P_0_ had the lowest stress response, C_3_P_3_ was intermediate, and C_3_P_6_ exhibited the highest stress‐bearing capacity. Notably, C_3_P_6_ had the highest fracture strain, indicating it could endure the largest deformation before failure, whereas the fracture strain of C_3_P_0_ was the highest and that of C_3_P_6_ the lowest.

To obtain more quantitative and statistical analysis, parallel samples were tested (Figure 2e,f). The results showed that with increasing PEGDA content, the fracture strain decreased by 60.3%, from 52.4% (C3P0) to 20.8% (C3P6). In contrast, the Young's modulus exhibited a 6.7‐fold increase, rising from 13.9 kPa (C3P0) to 93.1 kPa (C3P6). This enhancement in stiffness is primarily attributed to the higher crosslinking density introduced by PEGDA, which forms a more tightly connected polymer network, thereby improving the material's resistance to deformation under stress. The results showed that as the PEGDA content increased, the fracture strain decreased significantly from 52.4% for C3P0 to 20.8% for C_3_P_6_, and the Young's modulus increased progressively from 13.9 kPa for C_3_P_0_ to 93.1 kPa for C_3_P_6_. This trend can be attributed to the crosslinking density mechanism. As a crosslinker, PEGDA increases the number of C=C double bonds participating in the photo‐polymerization reaction with higher content, leading to a denser 3D network structure. This restricts polymer chain mobility, enhancing rigidity and modulus, but over‐crosslinking also reduces deformation ability, lowering the fracture strain.

Although C_3_P_0_ had the highest fracture strain, its low Young's modulus made it unsuitable for the mechanical strength required for microneedle skin penetration. Conversely, C_3_P_6_'s high modulus was accompanied by an undesirable fracture strain, indicating increased brittleness and potential mechanical mismatch with soft tissues, which could cause discomfort or microneedle breakage. In contrast, C_3_P_3_ offered a better balance between Young's modulus and fracture strain. Based on this, C_3_P_3_ was chosen as the optimized microneedle formulation for subsequent experiments.

**FIGURE 3:**
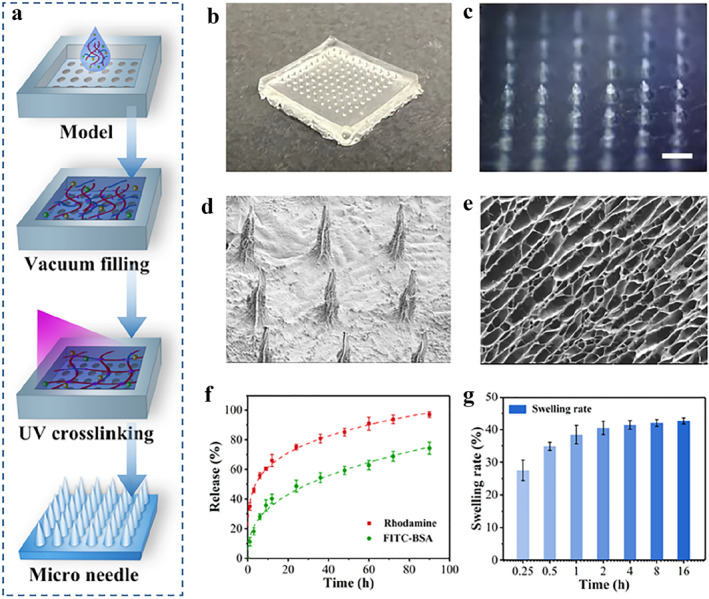
Preparation and characterization of microneedles. (a) Fabrication method. (b) Digital images. (c) Images under a stereomicroscope. (d) Arrays under SEM. (e) Porous network structure of microneedles under SEM. (f) Release profiles of two model drugs. (g) Swelling curves of microneedles.

Microneedles were fabricated using template molding. As shown in Figure 3a, polydimethylsiloxane (PDMS) was poured into a precisely machined positive mold (Supporting Information S1: Figure S2) and cured to create a negative mold with ordered conical grooves. VEGF and AMP were dispersed in PBS and then mixed with CS‐MA and PEGDA to prepare a pre‐gel solution. The drug‐loaded pre‐gel was injected into the negative mold and fully penetrated into the grooves under a vacuum. Finally, the mold was exposed to UV light, crosslinking the pre‐gel into hydrogel microneedles.

Figure 3b,c show the hydrogel microneedles stripped from the mold, revealing a conical array structure, which is conducive to skin penetration and drug delivery. The increased specific surface area also facilitates substance exchange. Figure 3d,e display SEM images of the hydrogel microneedles after freeze‐drying, showing a regular square array with uniform distribution and a porous internal structure. This high‐specific‐surface‐area and porous structure not only provides ample space for efficient drug loading but also enables capillary absorption of wound exudate, maintaining a moist healing environment. Moreover, the pores endow the microneedles with dynamic substance‐exchange capabilities supporting drug diffusion gradients and exudate management.

In vitro drug‐release experiments were done with rhodamine and FITC‐BSA as model drugs to simulate the release of small and large molecules. Results show both can be released from microneedles but with different kinetics. Rhodamine releases quickly, reaching over 60% in 12 h. FITC‐BSA releases slowly, entering a sustained‐release phase after 24 h, reaching about 72% in 96 h (Figure 3f). This biphasic kinetic is due to the different steric hindrances and diffusion abilities of large and small molecules. The initial burst release observed in Figure 3f is likely due to the rapid diffusion of surface‐adsorbed or loosely bound Rhodamine B and FITC‐BSA. The subsequent plateau phase reflects the slower, sustained release of drugs entrapped within the hydrogel matrix, governed by diffusion and matrix degradation. This biphasic release profile is common in hydrogel‐based systems and demonstrates the material's potential for controlled drug delivery.

With this design, the smaller AMP (∼1.5 kDA) is expected to release rapidly in the early treatment stage to quickly inhibit infection. The larger VEGF (∼40 kDA) is expected to release slowly and sustainably, acting during the repair phase to guide neovascularization. This release pattern avoids the local concentration toxicity or insufficient efficacy caused by burst release in traditional dressings, achieving appropriate temporal control of the wound‐healing process.

Additionally, the swelling properties of microneedles were studied (Figure 3g). Results show that drug‐loaded microneedles swell rapidly to 40% within 2 h of contact with body fluids and maintain a stable equilibrium. This moderate swelling enables dynamic absorption of tissue exudate while avoiding adverse effects of excessive swelling. These substance‐exchange capabilities ensure good interaction with biological tissues, laying the foundation for wound healing.

**FIGURE 4:**
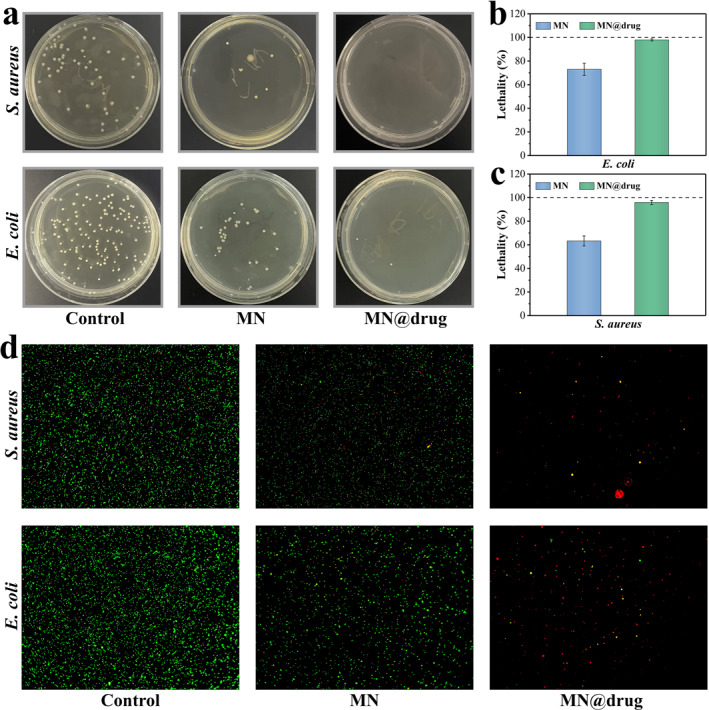
Antibacterial activity of microneedles. Bacteria interacted with microneedles during culture. Groups were divided into control, MN, and MN@drug groups. (a) Spread‐plate assay. (b–c) corresponding quantitative statistics with control as baseline to record lethality of the other two groups. (d) bacterial fluorescence staining results (live bacteria in green, dead in red).

To clarify the dual‐antibacterial mechanism of the microneedle system, which combines the inherent antimicrobial properties of the chitosan matrix and the active bactericidal action of AMP, we assessed the antibacterial effects of microneedles on *S. aureus* and *E. coli* using colony‐counting methods and live/dead staining (Figure 4a). The experimental groups were Control, MN, and MN@drug. Plate counting showed MN significantly suppressed both bacteria, reducing colony‐forming units (CFU) compared to the control. MN@drug had even better effects, with almost no colonies.

Quantitative analysis showed that MN inhibited *Staphylococcus aureus* and *Escherichia coli* by 73.0% and 63.3%, respectively. This was due to the protonated amino groups in chitosan adsorbing onto the negatively charged bacterial membrane via electrostatic interactions disrupting membrane potential and causing intracellular content leakage (Figure 4b,c). MN@drug had over 95% inhibition rates. The AMP molecules, with amphipathic structures were inserted into bacterial lipid bilayers, creating transmembrane ion channels. This caused osmotic imbalance and metabolic disruption, leading to bacterial lysis.

The SYTO 9/PI double‐staining system was used for live/dead staining verification (Figure 4d). Live bacteria with intact membranes fluoresce green, while dead bacteria with damaged membranes fluoresce red. Results showed Control had dense bacterial colonies with high green fluorescence; MN reduced bacterial counts with mixed red/green fluorescence; and MN@drug had few bacteria, mostly red fluorescence, indicating a low proportion of live bacteria. These results show the dual‐antibacterial mode of the microneedle system through the synergy of chitosan and AMP, offering efficient protection for infected wounds.

**FIGURE 5:**
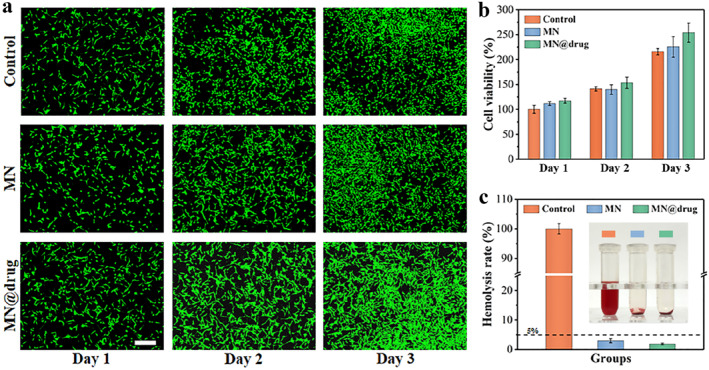
Biocompatibility assessment of microneedles. Groups were divided into control, MN, and MN@drug groups. (a) Live/dead fluorescence staining of NIH‐3T3 fibroblasts cultured with microneedle extract for 3 days (b) CCK‐8 assay results. There was no statistically significant difference in biocompatibility between the experimental groups and the control group, demonstrating the favorable biocompatibility of the material. (c) In vitro hemolysis test results.

Biocompatibility of microneedles is crucial for clinical application as they contact skin tissue long‐term. We assessed the safety of drug‐loaded microneedles through cell and blood compatibility tests. In cell compatibility tests, NIH‐3T3 fibroblasts, relevant to skin repair, were cultured in medium with microneedle extract. Live/dead fluorescence staining (Figure 5a) showed uniform cell distribution in both control and microneedle groups after 3 days, with cells having spread morphology and clear lamellipodia, and no dead cells observed. CCK‐8 tests further confirmed no significant difference in proliferation between groups, with viability exceeding 230% of the initial value after 3 days (Figure 5b), indicating no cytotoxicity of microneedle materials.

For blood compatibility, in vitro hemolysis tests evaluated the interaction between microneedle materials and blood components. Results showed that even at high concentrations (200 mg/mL), extracts from both drug‐loaded and unloaded microneedles caused no significant hemolysis, with hemolysis rates below 0.5% (the international safety threshold is 5%), proving their safety when contacting blood (Figure 5c). In summary, the microneedle system has excellent cell affinity and blood compatibility, establishing a biological safety foundation for its clinical application in infected wound repair.

**FIGURE 6:**
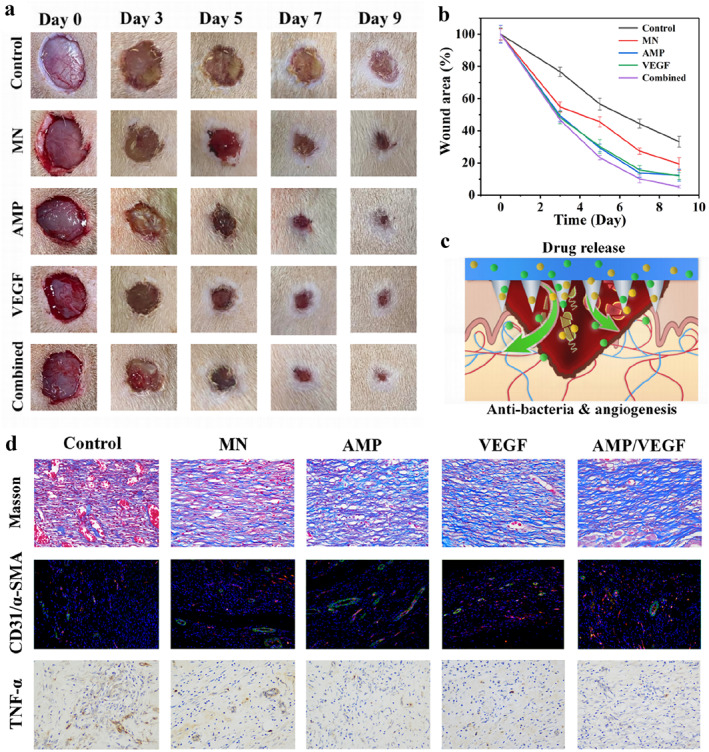
In vivo repair efficacy of microneedles in an infected rat wound model. (a) Gross morphology of wounds. (b) Dynamic changes in wound area over time. (c) Schematic of the wound‐healing process. (d) Immunofluorescence and histochemical characterization of wound tissues.

Profiting from the above physicochemical characterization, we systematically evaluated the therapeutic efficacy of microneedles in an infectious full‐thickness skin defect rat model. To delineate the functional contributions of distinct components, five experimental groups were established: PBS‐treated group (Control), blank drug‐free MN group (MN), AMP‐loaded MNs (AMP), VEGF‐loaded MNs (VEGF), and dual drug‐loaded MNs (Combine). Sequential wound documentation on post‐operative days 0, 3, 5, 7, and 9 revealed that all MN‐treated groups exhibited accelerated wound closure compared to the PBS control (Figure 6a). This confirmed the intrinsic advantages of the MN platform, attributable to its extracellular matrix‐mimetic soft‐wet properties and inherent antibacterial capacity. Notably, the Combine group demonstrated superior regeneration outcomes, including visible neogenic hair follicles in late‐stage healing (Figure 6a). Quantitative analysis further highlighted that the MN+combo group achieved the highest 12‐day healing rate, suggesting a synergistic interplay between AMP and VEGF (Figure 6b,c). This synergy likely stems from their complementary mechanisms: AMP‐mediated bacterial clearance eliminated pathogenic interference and established a sterile microenvironment for regeneration, while VEGF‐driven angiogenesis and collagen remodeling directly potentiated structural restoration. The Combine group thus concurrently addressed infection control and tissue reconstruction, achieving dual‐axis enhancement of the repair cascade.

To probe the histological underpinnings of healing progression, day‐12 wound tissues were subjected to multiparametric staining. Masson's trichrome staining (Figure 6d) demonstrated enhanced collagen deposition across all MN‐treated groups relative to the control, with the Combine group showing denser, more organized collagen fibrils—a critical indicator of biomechanical maturation. Dual immunofluorescence staining for CD31 (endothelial marker, red) and α‐SMA (vascular smooth muscle marker, green) (Figure 6d) vascular regeneration dynamics: sporadic neovascularization was observed in the PBS group; the MN + AMP group preserved endothelial integrity by mitigating infection‐induced vascular damage; the MN + VEGF group markedly expanded CD31/α‐SMA‐positive areas via VEGF‐driven endothelial activation; and the Combine group synergistically combined antibacterial protection with angiogenic stimulation, yielding the highest neovessel density and maturity. Immunohistochemical analysis of interleukin‐6 (IL‐6) (Figure 6d) further corroborated attenuated inflammatory responses across treatment groups, with the Combine group exhibiting the most pronounced suppression of IL‐6—a hallmark of its dual‐action efficacy. Although any exogenous peptide or protein theoretically carries a risk of immunogenicity, both AMP and VEGF used in this study are generally considered to have low immunogenic potential. The AMP selected shares high sequence homology with endogenous host defense peptides, reducing the likelihood of being recognized as a foreign antigen. VEGF, as a naturally occurring human growth factor, has been widely studied and applied in biomedical contexts with good safety profiles, particularly when locally and sustainably delivered. In our current in vitro and in vivo experiments, no signs of immune‐related adverse effects were observed. Nonetheless, we acknowledge the importance of this issue and plan to conduct more comprehensive immunological assessments in future studies.

## 3 Conclusion

In this study, we propose a drug‐loaded microneedle based on a copolymer hydrogel composed of methacrylated chitosan and polyethylene glycol diacrylate incorporating antimicrobial peptide and vascular endothelial growth factor. These microneedles were applied to wounds where their degradation facilitated the release of the loaded drugs to exert antibacterial and angiogenic effects. In vitro experiments demonstrated that our microneedles exhibit uniform morphology, good structural integrity, controlled drug release, and other excellent properties. Upon interaction with cells and bacteria, they displayed biocompatibility and superior dual antibacterial capabilities. In an in vivo infectious wound model, the microneedles significantly promoted wound healing through their antibacterial and angiogenic effects, showing clear advantages over the control group. Thus, these drug‐loaded microneedles serve as a multifunctional dressing, offering a promising novel strategy for wound repair.

In addition to demonstrating excellent in vitro and in vivo performance, our microneedle platform holds strong potential for clinical translation. The hydrogel‐based microneedle system is fabricated through a mild and scalable photopolymerization process, which is compatible with large‐scale production. The use of biocompatible and biodegradable components such as methacrylated chitosan and PEGDA ensures safety for clinical use. The microneedle fabrication process is compatible with scalable molding techniques and offers good batch‐to‐batch reproducibility. In addition, the hydrogel matrix provides a protective environment for loaded AMP and VEGF, supporting short‐term stability and laying the foundation for future long‐term preservation strategies. Moreover, the ability to co‐deliver both antibacterial and pro‐angiogenic agents directly into the wound bed makes this platform particularly suitable for the treatment of complex and chronic wounds including diabetic ulcers and infected skin injuries. Furthermore, the modular and biocompatible nature of this microneedle system makes it well‐suited for other chronic wounds, such as pressure ulcers and radiation‐induced injuries, which also involve impaired vascularization, persistent inflammation, and high infection risk. By tuning the drug combinations or release profiles, the platform can be readily adapted to meet the specific pathological needs of different wound types. These features suggest that our dual‐functional microneedle system is not only effective in preclinical models but also adaptable for future clinical applications.

## Author Contributions

F.K.B., Y.Y.Z., and W.J.S. conceived the idea and designed the experiment, J.Y.Z. conducted experiments and data analysis, J.Y.Z., Y.J.S., Y.X.Z., Z.J.F., and Y.C.Z. wrote the manuscript and participated in the scientific discussion.

## Conflicts of Interest

The authors declare no conflicts of interest.

## Supporting information

Supporting Information S1

## Data Availability

All data are available in the main text or the supplementary materials.
