# Supplementary material for: Antibacterial and Proangiogenic Hydrogel Microneedle Patches for Wound Healing
Source: Smart Med. 2025 Sep 9;4(3):e70014. doi: 10.1002/smmd.70014 (PMC12442652; doi:10.1002/smmd.70014)
Supplement: Supplementary file 1 — Supporting Information S1 [file SMMD-4-e70014-s001.docx]

**Supporting Information**

**Antibacterial and Proangiogenic Hydrogel Microneedle Patches for Wound Healing**

Junyi Zhang^1^, Yunjie Shi^1^, Yixin Zhang^1^, Zhiju Fang^1^, Yechao Zhou^1^, Feika Bian^1,^*, Yuyang Zhang^2,^*, Weijian Sun^1,^*

1. Department of Colorectal and Anal Surgery, The First Affiliated Hospital of Wenzhou Medical University, Wenzhou, Zhejiang, 325000, China

2. Department of Gynecology, The First Affiliated Hospital of Wenzhou Medical University, Wenzhou, 325000, China.

Email: bianfeika@wmu.edu.cn, zhangyuyang@wmu.edu.cn, weijiansun@wmu.edu.cn

**1. Experimental Section**

**Methods**

*Materials*

CS-MA was synthesized using chitosan and methacrylic anhydride (Sigma-Aldrich). Polyethylene glycol diacrylate (PEGDA, Mn = 700 Da), photoinitiator Irgacure 2959, antimicrobial peptide (AMP, TET-213), and vascular endothelial growth factor (VEGF165) were procured from MedChemExpress, China. *S. aureus* (ATCC 6538), *E. coli* (ATCC 25922), and NIH-3T3 fibroblasts were obtained from the China Center for Type Culture Collection. All animaexperiments were conducted in strict accordance with the recommendations in theGuide to the Care and Use of Experimental Animals.The Animal Experiment Ethics Committee of Wenzhou Institute of University of Chinese Academy of Sciences reviewed and approved all animal care and experimental protocols. The approval number is WIUCAS25051505.

*Synthesis of Methacrylated Chitosan*

Chitosan (3% w/v) was dissolved in 2% acetic acid under magnetic stirring. Methacrylic anhydride (40 mol% per glucosamine unit) was added dropwise under nitrogen atmosphere for 12 hours. The product was dialyzed (14 kDa) against deionized water for 72 hours, lyophilized, and characterized by FTIR. Successful methacrylation was confirmed by the appearance of C=C stretching vibrations.

*Fabrication of* *Drug-Loaded Microneedles*

A photocrosslinkable pre-gel solution was prepared by dissolving CS-MA (3% w/v) and PEGDA (0-6% w/v) in ultrapure water containing 0.1% Irgacure 2959. For drug-loaded microneedles, AMP (1 mg/mL) and VEGF (0.25 μg/mL) were uniformly dispersed into the pre-gel. The mixture was degassed under vacuum and injected into polydimethylsiloxane (PDMS) molds with conical cavities. After vacuum-assisted filling, the molds were exposed to 365 nm UV light (10 mW/cm², OmniCure S2000) for 120 seconds to induce crosslinking. Demolded microneedles were stored at 4°C in light-protected containers.

*Mechanical Characterization*

Uniaxial compression tests were performed on cylindrical hydrogel samples (6 mm diameter × 3 mm height) using an Instron 5944 universal tester at 1 mm/min strain rate.

*Swelling kinetics*

Swelling kinetics were evaluated by immersing microneedles in PBS (37°C) and measuring mass changes at predetermined intervals.

*SEM Morphological analysis*

SEM Morphological analysis was conducted via scanning electron microscopy (SU8010, Hitachi) after freeze-drying and gold sputter-coating.

*In Vitro Drug Release Profiling*

Dual-drug release kinetics were monitored using rhodamine B and FITC-BSA as model compounds. Microneedles were immersed in PBS (37°C) with periodic sampling. Released drug concentrations were quantified using a Varioskan LUX microplate reader (Thermo Fisher).

*Antibacterial Activity Assessment*

*S. aureus* and *E. coli* suspensions (10⁶ CFU/mL in LB broth) were incubated with microneedle patches (0.5 cm²) for 2 hours at 37°C. Bacterial viability was quantified by colony counting on agar plates and live/dead fluorescence staining (SYTO 9: 5 μM, PI: 30 μM; Keygen Biotech). Fluorescence images were acquired using a ZEISS Axio Vert.A1 microscope.

*Biocompatibility Evaluation*

Cytocompatibility was assessed by culturing NIH-3T3 fibroblasts in microneedle extracts (200 mg/mL) for 72 hours. Cell viability was measured via CCK-8 assay (Thermo Fisher) and live/dead staining (Calcein-AM/PI). Hemocompatibility was tested by incubating microneedle extracts with fresh rat blood (4% v/v) for 1 hour, with hemolysis percentage calculated from absorbance.

*Ethical Approval*

All animal procedures were performed in strict accordance with institutional guidelines and the ethical standards of animal care. The experimental protocol was reviewed and approved by the Animal Experiment Ethics Committee of Wenzhou Institute of University of Chinese Academy of Sciences (Approval No. WIUCAS25051505). All efforts were made to minimize animal suffering, including the use of appropriate anesthesia and humane endpoints. Animal housing, care, and surgical procedures were conducted in compliance with the 3R principles (Replacement, Reduction, and Refinement) to ensure ethical and responsible use of laboratory animals throughout the study.

*Animal experiment*

All animal experiments were approved by the Animal Experiment Ethics Committee of Wenzhou Institute of University of Chinese Academy of Sciences (Approval No. WIUCAS25051505). Twenty-five male Sprague-Dawley rats (220–250 g) were randomly assigned into five groups (n = 5 per group): Control (PBS), blank MN, AMP-MN, VEGF-MN, and AMP+VEGF-MN.

An infectious full-thickness skin defect model was established as follows: after anesthesia, a full-thickness wound was surgically created on the dorsal skin of each rat under aseptic conditions. Immediately after injury, 100 µL of *S. aureus* suspension (10⁸ CFU/mL) was applied to the wound to induce localized infection. Treatments were applied topically 12 hours post-infection.

Animals were randomly assigned using a computer-generated list after acclimation. All wound size measurements, gross imaging, and histological assessments were conducted in a blinded manner by independent investigators.

*Statistical Analysis*

Data are presented as mean ± standard deviation. One-way ANOVA with Tukey’s post-hoc test was applied using GraphPad Prism 9.0. Significance was defined as *p < 0.05, **p < 0.01, ***p < 0.001.

**2. Supporting Figures**

**
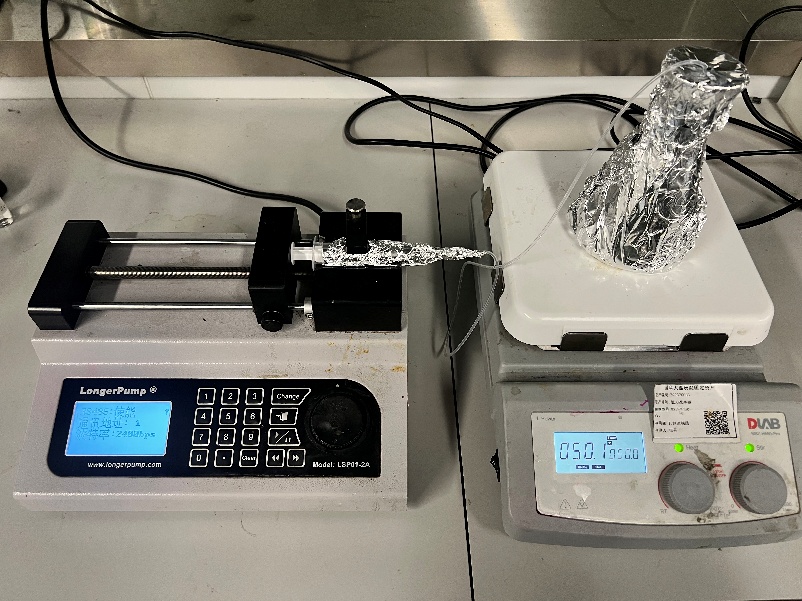
Figure S1.** Photographic illustration of the experimental setup used for the fabrication of microneedle molds. The system comprises a programmable syringe pump (LongerPump LSP01-2A) and a magnetic stirring hotplate to enable precise precursor injection and controlled thermal reaction conditions. The microneedle molds were fabricated using a standard SU-8 photolithographic master as the template.


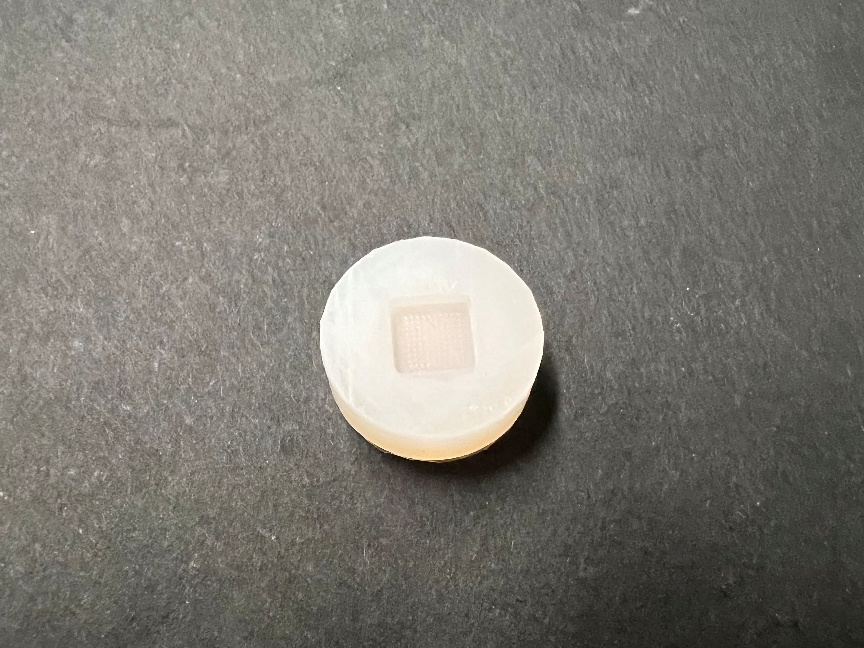


**Figure S2.** Representative image of a microneedle patch fabricated from chemically synthesized chitosan methacrylate (CSMA) hydrogel. CSMA was prepared via methacrylation of chitosan, followed by photocrosslinking to produce a mechanically stable microneedle structure with uniform geometry and well-defined morphology.
